# Proteomic Characterization of Primary Human Pancreatic Cancer Cell Lines Following Long-Term Exposure to Gemcitabine

**DOI:** 10.3390/proteomes13040048

**Published:** 2025-10-01

**Authors:** Manoj Amrutkar, Yuchuan Li, Anette Vefferstad Finstadsveen, Caroline S. Verbeke, Ivar P. Gladhaug

**Affiliations:** 1 Department of Pathology, Division of Laboratory Medicine, Oslo University Hospital, 0424 Oslo, Norwayc.s.verbeke@medisin.uio.no (C.S.V.); 2 Institute of Clinical Medicine, Faculty of Medicine, University of Oslo, 0316 Oslo, Norway

**Keywords:** pancreatic cancer, proteomics, mass spectrometry, gemcitabine sensitivity

## Abstract

Background: Gemcitabine (GEM) remains a cornerstone in the treatment of pancreatic cancer. Upon exposure to GEM, pancreatic cancer cells (PCCs) tend to adapt quickly to outcompete drug-induced cytotoxicity, thereby contributing to treatment failure. Thus, understanding GEM-induced molecular changes in PCCs is important. Methods: Three primary PCC lines (PCC-1, PCC-2, PCC-7) and Mia PaCa-2 cultured for 40 passages (p) in the absence (control) or presence of GEM (GemR) were assessed for phenotypic changes. Proteome profiles for all PCCs at p10, p20, p25, p30, p35, and p40 were obtained using mass spectrometry (MS). Protein expression was determined using immunoblotting. Differentially abundant proteins (DAPs) were evaluated for enrichment of functional and biological attributes and protein–protein interactions. Results: GEM sensitivity and growth were both reduced in GemR versus paired controls for all four PCC lines. MS mapped > 7000 proteins in each PCC line, and the abundance of 70–83% of these was found to be significantly altered when comparing all sample groups. Proteomic changes in GemR versus paired controls differed remarkably among the PCCs and were affected by passaging and treatment duration. DAPs at p40 were mostly related to metabolic pathways, including nucleotide metabolism and diverse cell growth processes. Several closely related DAPs and multiple hub proteins in each PCC line were identified. Conclusions: Overall, this study revealed cell-line-specific, heterogeneous changes in proteome profiles of PCCs following their long-term exposure to GEM, and these were likely affected by treatment duration, dosage, and passaging.

## 1. Introduction

Pancreatic ductal adenocarcinoma (PDAC), the most frequent malignancy of the pancreas, is a solid tumor with an overall poor prognosis and a 5-year survival of less than 13%. PDAC is projected to become the second leading cause of cancer-related death in the Western world by 2030 [1,2]. Chemotherapy remains a cornerstone of PDAC management; almost all PDAC patients are offered chemotherapy either as primary treatment or as part of a multimodal approach alongside surgery or radiation therapy [3]. The currently available PDAC chemotherapy options include gemcitabine (GEM) monotherapy and two combination regimens—FOLFIRINOX [folinic acid (leucovorin calcium), fluorouracil, irinotecan, and oxaliplatin] and GEM plus nab-paclitaxel (Abraxane) [4,5]. GEM has remained crucial for PDAC treatment strategies since its first demonstrated survival benefits in patients with advanced pancreatic cancer in 1997 [6]. FOLFIRINOX is reported to be more effective but also less tolerable due to high toxicity. Hence, GEM alone or in combination with nab-paclitaxel is the preferred option for PDAC patients with less tolerance to treatment-related side effects [7,8].

GEM (2′,2′-difluoro-2′-deoxycytidine, dFdC) is a synthetic nucleoside analog prodrug that requires uptake/transport and intracellular activation to induce its cytotoxic effect [9,10,11]. Upon exposure to GEM, pancreatic cancer cells (PCCs) tend to adapt quickly by altering molecular signaling pathways to outcompete drug-induced cytotoxicity to maintain their growth. These include changes in the cell state that reduce or block drug transport, increase detoxification, and reduce activation of GEM [10,12,13]. Such adaptation by PCCs is achieved by epigenetic modifications, metabolic reprogramming, and factors from the pancreatic tumor microenvironment (TME), particularly cancer-associated fibroblasts (CAFs), tumor-associated macrophages, and extracellular matrix (ECM) components [14,15,16,17,18]. These changes subsequently lead to treatment failure, as tumors stop responding to the cytotoxic actions of GEM, and it may also cause multi-drug resistance, limiting the use of available alternative chemotherapy options [19,20].

Extensive research carried out during the past two decades to investigate the causes of treatment failure in PDAC revealed the multi-faceted nature of GEM resistance and identified diverse factors that contribute to reduced GEM sensitivity in PDAC [10,11,21,22]. These include both cell-intrinsic modifications that facilitate cancer cells to survive in the presence of cytotoxic agents, as well as non-cell autonomous mechanisms of resistance, such as the impact of the TME. The pancreatic TME accounts for up to 80% of the tumor mass and consists of an abundant ECM, nonmalignant cells (CAFs, immune cells, adipocytes, endothelial cells), and other components such as growth factors and cytokines [10,11]. However, the current limited understanding of the molecular mechanisms underlying therapy resistance in PDAC significantly hinders the development of strategies to overcome this challenge. Moreover, there is no clear data that describes the molecular changes that occur in PDAC cells during long-term exposure to GEM. To this end, proteomic profiling seems an ideal approach, as it has recently been used for detecting and validating protein biomarkers and therapeutic targets for GEM response in PDAC [17,23,24,25].

In this study, PCCs collected at several time points during their maintenance in the medium with or without GEM for 40 passages were characterized for changes in proteome profiles using data-independent acquisition (DIA)–mass spectrometry (MS). DIA-MS has emerged as a most powerful, high-throughput proteomics technique that provides quantification accuracy and repeatability [26,27]. The main aim of this study was to investigate proteomic changes accompanying long-term GEM exposure with the purpose of characterizing molecular changes associated with the timeline during which PCCs are likely to reduce GEM sensitivity.

## 2. Materials and Methods

### 2.1. Cell Culture

Four human PCC lines were used in this study, including three primary—PCC-1, PCC-2, and PCC-7, obtained in-house at the Oslo University Hospital, Norway—and an immortalized Mia PaCa-2 (American Type Culture Collection; Manassas, VA, USA). PCC-1, PCC-2, and PCC-7 were established from three resected treatment-naïve PDACs of a 67-year-old woman, a 62-year-old man, and a 73-year-old woman, respectively, using the outgrowth method [28]. All PCCs were cultured and maintained at 37 °C with 5% CO_2_ in normal growth medium (NGM), i.e., Dulbecco’s modified Eagle’s medium (DMEM) containing 4.5 g/L glucose (GlutaMAX, #31966047; Thermo Fisher Scientific, Waltham, MA, USA) supplemented with 10% fetal bovine serum (FBS; #10500064; Thermo Fisher Scientific), 1% penicillin–streptomycin (#15140122; Thermo Fisher Scientific), and 1% amphotericin B (#15290026; Thermo Fisher Scientific). Cell cultures were routinely checked for mycoplasma using MycoAlert mycoplasma detection kit (#LT07-703; Lonza, Basel, Switzerland). Short tandem repeat analysis was used for cell authentication (Eurofins Genomics; Ebersberg, Germany).

### 2.2. Long-Term Exposure to Gemcitabine

All four PCC lines were maintained for a total of 40 passages in 100 mm cell culture dishes in NGM without (control group) or with GEM (GemR group). For the GemR group, PCCs were incubated with GEM (G6423; Sigma–Aldrich, St. Louis, MO, USA) at a final concentration of 50 nM for the first 25 passages and 150 nM for the following 15 passages. Cells were passaged upon reaching >70% confluence. Aliquots of both GemR and control cells were collected at the following passages: p10, p20, p25, p30, p35, and p40. Cells were cryopreserved and stored until further use following the standard protocol. Total duration required for the individual PCC line to reach p40 for control/GemR was as follows: PCC-1—6.3/7.2 months, PCC-2—6.2/7.3 months, PCC-7—6.3/8.1 months, and Mia PaCa-2—6.2/7.8 months.

### 2.3. Gemcitabine Sensitivity

Both GemR and control PCCs at p40 were seeded in 96-well plates at a density of 5000 cells per well in 100 µL NGM, in four replicates. The following day, cells were incubated for 72 h with growth medium containing GEM at a final concentration range of 0.01–100 µM or with DMSO for the untreated control. GEM-induced cytotoxicity was determined using the CellTiter-Glo luminescent cell viability assay kit (G7570; Promega, Madison, WI, USA).

### 2.4. Cell Morphology and Growth

Seeding density was 10,000 cells per well in 500 µL NGM in 24-well plates for morphology and viability experiments and 2000 cells per well in 100 µL NGM in 96-well plates for proliferation experiments. Cells were seeded in four replicates for each condition. Cell morphology changes were determined by visual inspection under the light microscope, and representative images were taken. For viability experiments, cells were seeded in parallel in two separate plates and incubated for 24 h and 96 h. At the respective time points, the cell number was counted using the Invitrogen Countess Automated Cell Counter (Thermo Scientific, Waltham, MA, USA). For proliferation assessment, cells were incubated for 96 h, and the incorporation of BrdU was determined using the BrdU cell proliferation ELISA kit (ab126556; Abcam, Cambridge, UK).

### 2.5. Extracellular Lactate Content

A 10 µL aliquot of cell culture supernatant from each well was collected during viability experiments. The amount of lactate in the culture supernatants was determined using the glycolysis cell-based assay kit (#600450; Cayman Chemicals, Ann Arbor, MI, USA) according to the manufacturer’s instructions. Lactate amount was adjusted for the cell number of the individual well.

### 2.6. Proteomic Analysis

Cell lysates: Both control and GemR cells from all six different passages (p10, p20, p25, p30, p35, and p40) for all four PCC lines (PCC-1, PCC-2, PCC-3, and Mia PaCa-2) were seeded in triplicate, for each condition, in 6-well plates at a density of 100,000 cells per well in 2 mL NGM. Upon reaching >50% confluence, cells were harvested using RIPA buffer (#20-188; Millipore, Burlington, MA, USA; 160 µL/well) supplemented with protease and phosphatase inhibitors. Cell lysates were stored at −80 °C until analysis.

On-bead precipitation and protein digestion: For each replicate, an equal amount of 10 µg protein was precipitated with 70% acetonitrile onto magnetic beads (MagReSyn^®^ Amine; Resyn Biosciences, Edenvale, Gauteng, South Africa) [29]. The proteins were washed on the beads with 100% acetonitrile and 70% ethanol, and then resuspended in 50 µL ammonium bicarbonate (50 mM) containing 10 mM dithiothreitol (DTT) for reduction of cysteines. Samples were incubated at 37 °C for 40 min. Then, to alkylate proteins, 50 µL of 30 mM indole-3-acetic acid (IAA) in 50 mM ammonium bicarbonate was added, and samples were incubated at room temperature in the dark for 30 min. Trypsin (0.5 µg) was added to each sample for overnight on-bead protein digestion at 37 °C. The resulting peptides were concentrated and desalted on EVOTIPS for mass spectrometry (MS) analysis according to the standard protocol from EVOSEP Biosystems (Odense, Denmark). Each sample was analyzed as a single technical replicate.

Liquid chromatography–tandem mass spectrometry (LC-MS/MS): LC-MS/MS analysis was carried out using an EVOSEP one LC system (EVOSEP Biosystems, Odense, Denmark) coupled to a timsTOF Pro2 mass spectrometer using a CaptiveSpray nanoelectrospray ion source (Bruker Corporation, Bremen, Germany). Samples of 200 ng digested peptides were loaded onto a capillary C18 column (15 cm length, 150 μm inner diameter, 1.5 μm particle size; EVOSEP). Peptides were separated at 40 °C using the standard 30 sample/day method from EVOSEP. The timsTOF Pro2 mass spectrometer was operated in DIA-PASEF mode. Mass spectra for MS were recorded between *m*/*z* 100 and 1700. Ion mobility resolution was set to 0.85–1.30 V s/cm over a ramp time of 100 ms. The MS/MS mass range was limited to *m*/*z* 475–1000 and ion mobility resolution to 0.85–1.27 V s/cm to exclude singly charged ions. The estimated cycle time was 0.95 s with 8 cycles using DIA windows of 25 Da. Collisional energy was ramped from 20 eV at 0.60 V s/cm to 59 eV at 1.60 V s/cm.

Data processing: Raw mass spectrometry (MS) data files were submitted to data-independent acquisition proteomics data processing (DIA-NN 1.8.1) [30] for protein identification and label-free quantification (LFQ) using the library-free function. MS analysis and data processing were performed following the parameters described previously [31]. The UniProt human database (European Bioinformatics Institute, EMBL-EBI, Cambridge, UK) was used to generate a library in silico from a human FASTA file. Carbamidomethyl (C) was set as a fixed modification. Trypsin without proline restriction enzyme option was used, with one allowed miscleavage, and peptide length range was set to 7–30 amino acids. The mass accuracy was set to 15 ppm, and the precursor false discovery rate (FDR) allowed was 0.01 (1%).

Data analysis: MS data were further processed using Perseus 2.0.11.0 (Max-Planck Institute of Biochemistry, Martinsried, Germany). Qlucore Omics Explorer 3.8.22 (Qlucore AB, Lund, Sweden) was used for data visualization by principal component analysis (PCA) and generation of heatmaps [32,33]. The proteins with >30% difference in abundance between groups at *p* < 0.05 were considered differentially abundant proteins (DAPs).

Functional enrichment analysis: Enriched biological and functional attributes for DAPs were identified using the Database for Annotation, Visualization, and Integrated Discovery (DAVID) 6.8 [32]. The characteristic biological attributes were identified using gene ontology (GO) analysis, while the functional attributes were identified using Kyoto Encyclopedia of Genes and Genomes (KEGG) pathway enrichment analysis. A *p*-value < 0.05 was considered a statistically significant enrichment.

Protein–protein interaction (PPI) networks: To assess the functional interactions between proteins, the PPI networks were generated using STRING 12.0 [34] and were visualized using Cytoscape 3.10.2, cytoHubba plugin, by applying centrality parameters described previously [35]. Centrality parameters, including degree, closeness, and betweenness, were determined using the cytoHubba. The average number of interactions relevant to each node (proteins) was used as the degree of a given node. Nodes with a higher degree are considered as hub proteins. Degree centrality is the number of links to a given node, while betweenness centrality measures the number of shortest paths passing through a node within a network.

### 2.7. Immunoblotting

Protein expression was analyzed using immunoblotting, as described previously [33]. Briefly, cells grown to confluence in 6-well plates were lysed in RIPA lysis buffer containing proteinase and phosphatase inhibitors. Cell lysates were centrifuged, and the supernatant was collected before protein concentration was measured with Pierce BCA protein assay kit (#23225; Thermo Scientific, Waltham, MA, USA). Samples were diluted with the Laemmli buffer containing 50 mM DTT to a final concentration of 1 µg/µL. Proteins were separated by electrophoresis, transferred to nitrocellulose membrane, blocked, and subsequently incubated overnight with the respective primary antibodies, followed by 1 h incubation with HRP-conjugated Goat anti-Rabbit secondary antibody (1706515; Bio-Rad, Hercules, CA, USA). GAPDH and vinculin was used as a loading control. Blots were developed using SuperSignal Chemiluminescent reagent (Thermo Scientific, Waltham, MA, USA). Images were captured using ChemiDoc MP Imaging System 6.0 (Bio-Rad). Band intensities were analyzed using Image Lab Software 6.1 (Bio-Rad). Primary antibodies against the following targets were used: aldehyde dehydrogenase 1A1 (ALDH1A1; ab52492) and kallikrein-6 (KLK6; ab190924) from Abcam; mucosa-associated lymphoid tissue lymphoma translocation protein 1 (MALT1; #2494), 5′-nucleotidase (NT5E/CD73; #13160), glyceraldehyde-3-phosphate dehydrogenase (GAPDH; #5174), and vinculin (#13901) from Cell Signaling Technology (Danvers, MA, USA); tumor protein D53 (TPD52L1/hD53; #14732-1-AP) from Proteintech (Manchester, UK).

### 2.8. Statistical Analysis

The statistical analysis of the results was performed using GraphPad Prism 9 (GraphPad Software, CA, USA) or by an unpaired two-tailed Student’s *t*-test or ANOVA for multi-group comparison; *p* < 0.05 was considered statistically significant. Data are expressed as means ± SEM.

## 3. Results

### 3.1. GEM Sensitivity

**Figure 1:**
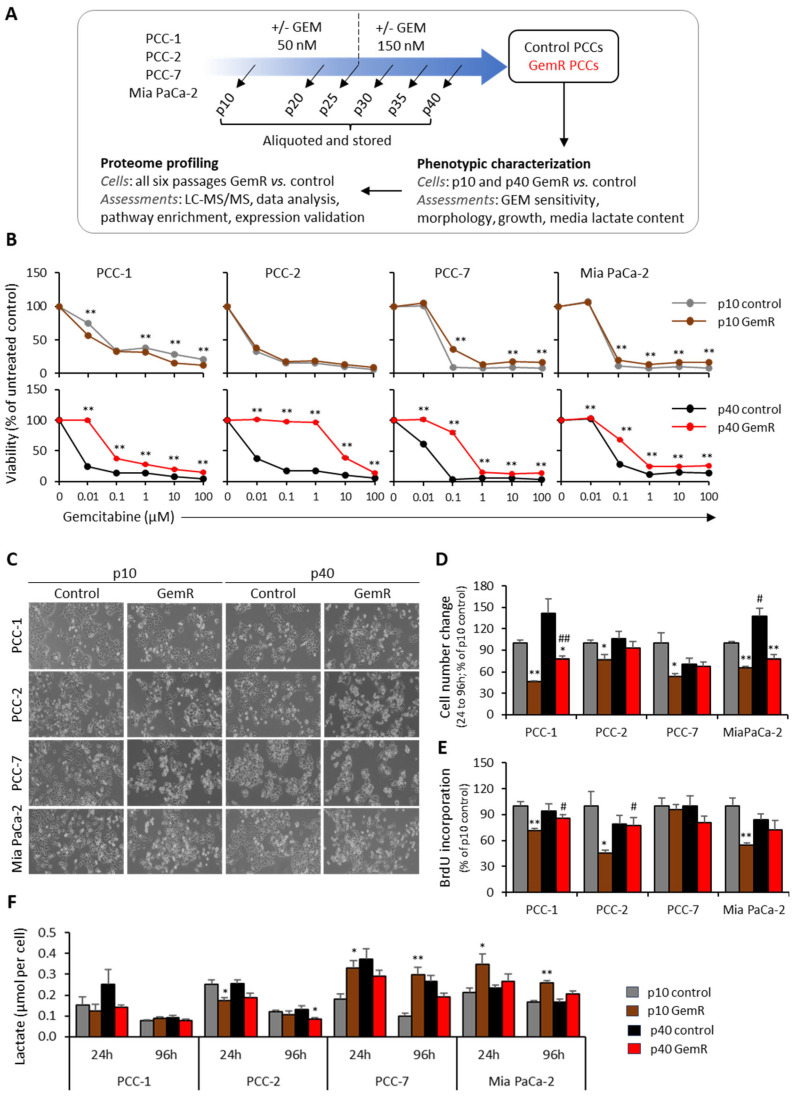
Study outline, phenotypic characterization. (**A**) Study outline including treatment scheme. (**B**–**F**) Phenotypic characterization of both GemR and paired control PCCs at p10 and p40. (**B**) PCCs’ viability evaluated using CellTiter-Glo assay following their exposure with indicated concentrations of GEM for 72 h; viability of PCCs treated with DMSO were set to 100%. (**C**) Representative cell morphology images captured around 72 h from seeding. (**D**) Relative change in cell number between 24 h to 96 h. (**E**) Relative proportion of actively proliferating cells determined by BrdU incorporation at 96 h. (**F**) Media lactate levels. Data are means ± SEM of four replicates and * *p* < 0.05, ** *p* < 0.01 comparing GemR versus control at p10 or p40. # *p* < 0.05, ## *p* < 0.01 comparing p40 versus p10 in both groups. GEM, gemcitabine; GemR, gemcitabine-treated cells; LC-MS/MS, liquid chromatography–tandem mass spectrometry; p, passage; PCCs, pancreatic cancer cells.

The study outline, including the GEM treatment scheme, is presented in Figure 1A. The PCCs’ sensitivity to GEM was evaluated by assessing their response in terms of change in viability following 72 h exposure to higher concentrations of GEM (0.01–100 µM). Cells at p10 displayed a somewhat diverse response, with GemR versus paired controls showing relatively higher GEM sensitivity for PCC-1, slightly less sensitivity for both PCC-7 and Mia PaCa-2, and a similar response in both groups for PCC-2 (Figure 1B). As anticipated, GEM sensitivity was significantly lower in GemR cells at p40 compared with paired controls for all PCC lines (Figure 1B). Notably, reduced GEM sensitivity was visible at concentrations as low as 0.01 µM for three primary PCCs and 0.1 µM for Mia PaCa-2 (Figure 1B).

### 3.2. Phenotypic Differences

The morphological differences between GemR and paired controls varied between the four PCC lines (Figure 1C). The GemR compared with paired controls at p40 appeared relatively small, circular, and were growing in smaller colonies, while no noticeable differences were seen at p10 (Figure 1C). Cell growth, determined by measuring cell numbers at 24 h and 96 h from the time of seeding, revealed a significantly lower growth in GemR versus paired controls for all PCCs at p10 (1.3–2.1-fold) and for PCC-1 and Mia PaCa-2 at p40 (1.8-fold; Figure 1D). Cell proliferation, assessed by measuring BrdU incorporation, was significantly lower in GemR compared with paired controls (1.4–2.2-fold) for three PCC lines (except PCC-7) at p10, while it was unchanged across PCCs at p40 (Figure 1E). Moreover, comparing controls at p40 versus p10 revealed significantly increased growth for Mia PaCa-2 only (1.4-fold; Figure 1D), while BrdU incorporation was unchanged across PCC lines (Figure 1E). Furthermore, the amount of lactate in the cell culture supernatants collected during growth experiments as an indirect measure of glycolysis was determined. It was generally lower at 96 h compared with 24 h but varied among the four PCC lines (Figure 1F). Comparing lactate content for GemR versus paired controls showed no significant change for PCC-1, significantly lower at 24 h (p10) and 96 h (p40) (1.4–1.5-fold) for PCC-2, and significantly higher only at p10 for both 24 h (1.8- and 1.7-fold) and 96 h (3.0- and 1.6-fold) for both PCC-7 and Mia PaCa-2 (Figure 1F).

### 3.3. Overview of Proteome Profiles

**Figure 2:**
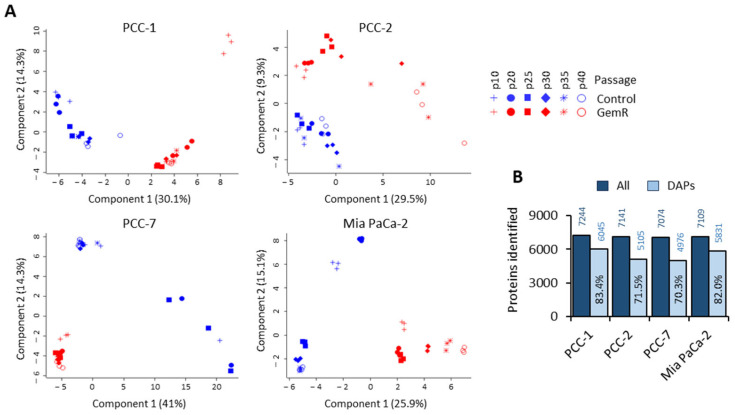
Overview of proteome profiles. Proteome profiles of GemR and paired control PCCs were obtained using liquid chromatography–mass spectrometry analysis of whole-cell lysates. (**A**) Separate principal component analysis (PCA) score plots for each PCC line. Each symbol represents the individual sample analyzed. (**B**) Overview of all proteins identified and the proportion of differentially abundant proteins (DAPs) between GemR versus paired control cells. GemR, gemcitabine-treated cells; p, passage; PCC, pancreatic cancer cell.

Whole-cell proteome profiles of both GemR and paired controls at six different time points (p10, p20, p25, p20, p35, and p40) for all PCC lines were obtained using LC-MS/MS. The proteome datasets are provided in Supplementary Materials File S1. The overall proteome data and its distribution among samples and between time points was visualized using unsupervised principal component analysis (PCA). The PCA plots demonstrate significant heterogeneity in proteome profiles between the four PCC lines as well as between various time points in each PCC line (Figure 2A). GemR and control groups appear to be mainly separated by component 1 for PCC-1 and Mia PaCa-2 and by component 2 for PCC-2 and PCC-7. Moreover, three replicate samples in each group were seen distributed homogenously in PCC-1 and Mia PaCa-2 and mostly heterogeneously in PCC-2 and PCC-7 (Figure 2A). Some control samples in PCC-7 displayed a noticeable intra-group variation. As such, one replicate in the PCC-7–p20 control group was removed from further analysis due to being identified as a significant outlier. MS mapped more than 7000 proteins in each PCC line, and a vast majority (70 to 83%) of these were significantly altered (*p* < 0.05; FC > 1.2) when we compared all sample groups in each PCC line (Figure 2B).

### 3.4. Differentially Abundant Proteins (DAPs) in GemR Versus Control Cells

**Figure 3:**
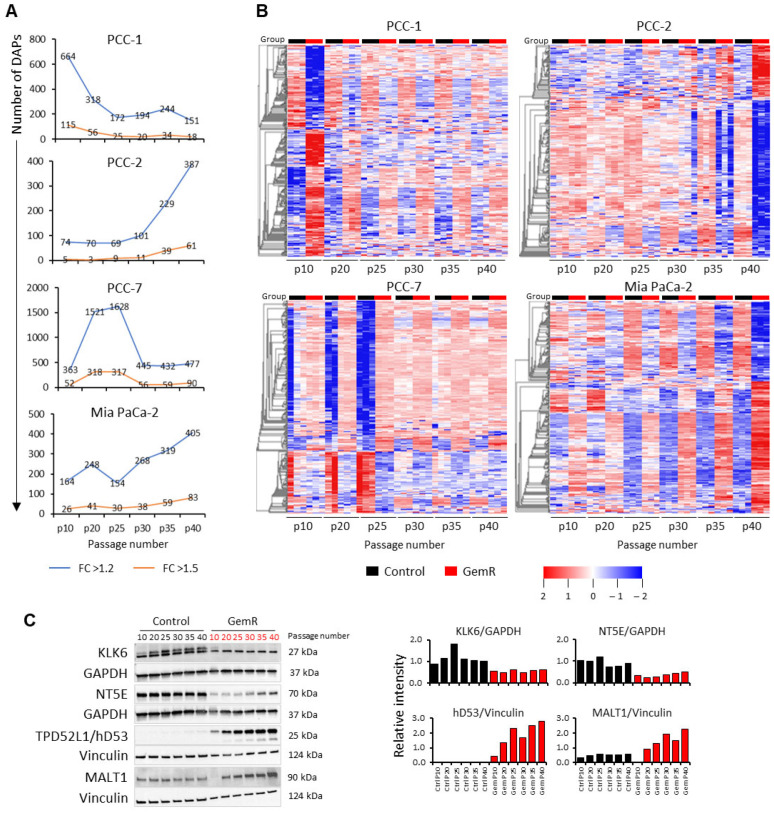
Differentially abundant proteins (DAPs) between GemR versus control cells. (**A**) Number of DAPs at each time point for the individual PCC lines. DAPs with difference of log2 fold change of >1.2 (blue line) and >1.5 (orange line) are presented separately. (**B**) Heatmaps for DAPs with maximum count, i.e., p10 in PCC-1 (664), p40 in PCC-2 (387) and Mia PaCa-2 (405), and p25 in PCC-7 (317), showing the pattern of their abundance at all time points in each PCC line. (**C**) Immunoblots showing the expression of indicated proteins including loading controls—GAPDH and vinculin. Expression levels were determined by calculating the band intensities of target proteins relative to loading controls. GAPDH, glyceraldehyde-3-phosphate dehydrogenase; GemR, gemcitabine-treated cells; KLK6, kallikrein-6; MALT1, mucosa-associated lymphoid tissue lymphoma translocation protein 1; NT5E, 5′-nucleotidase; p, passage; PCC, pancreatic cancer cell; TPD52L1 (hD53), tumor protein D53.

Next, the number of DAPs in GemR versus paired controls with *p* < 0.05 and FC > 1.2 or >1.5 at each time point for all four PCC lines were identified (Figure 3A, Supplementary Materials File S2). The number of DAPs at a given time point differed between the four PCC lines and was maximum at p10 in PCC-1, p25 in PCC-7, and p40 in PCC-2 and Mia PaCa-2 (Figure 3A). Interestingly, the time point-associated change in DAP count was mainly seen until p25 in PCC-1 and PCC-7, while it increased consistently from p25 and onward in both PCC-2 and Mia PaCa-2 (Figure 3A). Proteome profiles of the maximum number of DAPs with FC > 1.5 for PCC-7 (317 at p25) and with FC > 1.2 for the other three PCC lines at all time points are presented as heatmaps (Figure 3B). These revealed notable differences in the abundance of these DAPs, with the largest differences at p10 in PCC-1, p40 in PCC-2 and Mia PaCa-2, and p25 in PCC-7 (Figure 3B). Moreover, except for PCC-2, the abundance of most DAPs in the other three PCC lines was higher in GemR versus paired controls (Figure 3B). Furthermore, proteome profile alterations in Mia PaCa-2 were generally in line with time point changes, while they appeared as sudden events in primary PCCs, including the potential turning points p20 for PCC-1 and p25 for PCC-2 and PCC-7 (Figure 3A,B).

Searching DAPs common to multiple time points, each PCC line revealed 363, 207, 1371, and 381 DAPs common to at least two time points, and 20, 2, 51, and 25 of these were common to all six time points in PCC-1, PCC-2, PCC-7, and Mia PaCa-2, respectively. The pattern of their abundance is provided as heatmaps in Figure S1. Kallikrein-6 (KLK6) was the only DAP whose level was found altered at all time points in GemR versus controls in all PCC lines, although the direction of change differed among the PCC lines (Table 1, Figure S2A). Of the DAPs common to at least two time points in each PCC line, 13 were common to the three primary PCC lines: AFP, ALG5, ASB3, C1QTNF3, KLK6, MT-ATP6, NDUFA4, NUFIP1, PRR14, TMA7, TNIK, UFM1, and UXT, and 6 were common to PCC-2, PCC-7, and Mia PaCa-2: CCDC71L, IGFBP6, INPP4B, MALT1, PSMB9, TGM2 (Table 1, Figure S2B). Searching their potential known roles in databases UniProt and GeneCards revealed their involvement in diverse biological processes (Table 1).

For the technical validation of proteomic data, the expression of KLK6, NT5E, hD53, and MALT1 was assessed in Mia PaCa-2 cells using immunoblotting. Consistent with the proteomic data, immunoblotting confirmed significantly reduced expression of KLK6 and NT5E and significantly increased expression of hD53 and MALT1 in GemR versus control cells (Figure 3C and Figure S3).

### 3.5. Proteins Affected by GEM Exposure

**Figure 4:**
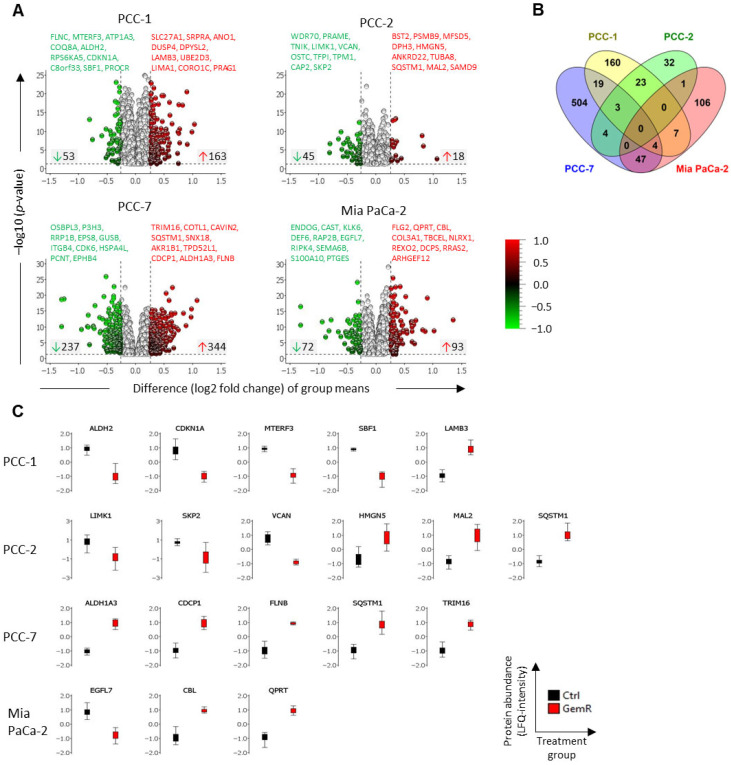
Overall altered proteome profiles between GemR versus control PCCs. (**A**) Volcano plots for each PCC line showing distribution of differentially abundant proteins (DAPs) with significantly altered overall proteome profiles in GemR versus paired untreated control PCCs (*p* < 0.05; FC > 1.2). Ten most up- and down-regulated DAPs in each PCC line are listed on each plot. (**B**) Venn diagram showing distribution of DAPs (from (**A**)) between four PCC lines. (**C**) Boxplots showing levels of DAPs reported to be involved in the regulation of gemcitabine sensitivity and/or cancer stemness. Values are log2-transformed label-free quantitation (LFQ) intensities obtained from mass spectrometry analysis. Data are presented as the average of all samples in each treatment group. GemR, gemcitabine-treated cells; PCC, pancreatic cancer cell.

First, DAPs with significantly altered overall proteome profiles (i.e., average of all time points) in GemR versus the control group (*p* < 0.05; FC > 1.2) were selected, resulting in 216, 63, 581, and 165 DAPs in PCC-1, PCC-2, PCC-7, and Mia PaCa-2, respectively. Their distribution is shown using volcano plots in Figure 4A. A total 108 of these DAPs were common to at least two PCC lines, while only 3 DAPs were common to all primary PCC lines: ASB3, RPL22L1, and UFM1 (Figure 4B). The twenty most altered DAPs in each PCC line (Figure 4A) were searched for their reported involvement in the regulation of GEM sensitivity and cancer stemness using the databases UniProt, GeneCards, and PubMed. This identified ALDH2, CDKN1A, LAMB3, MTERF3, and SBF1 in PCC-1; LIMK1, HMGN5, MAL2, SKP2, SQSTM1, and VCAN in PCC-2; ALDH1A3, CDCP1, FLNB, SQSTM1, and TRIM16 in PCC-7; CBL, EGFL7, and QPRT in Mia PaCa-2. Their levels are presented as boxplots in Figure 4C.

Second, to account for potential alterations in proteome profiles due to multiple passaging and longer maintenance of cells in culture, such as up to 40 passages in this study, the top 100 DAPs in each PCC line identified through multi-group comparison (shown in Figure 2B) were selected for further analysis. Of these, 80, 80, 90, and 85 DAPs in PCC-1, PCC-2, PCC-7, and Mia PaCa-2, respectively, were found altered at all time points in GemR versus controls (Table S1). Interestingly, the abundance of 39, 35, 21, and 15 DAPs in PCC-1, PCC-2, PCC-7, and Mia PaCa-2, respectively, was unchanged throughout 40 passages in control cells (Table S1), whereas 31, 23, 21, and 14 of these DAPs were significantly altered in GemR compared with the control group (Table 2). Notably, 12, 38, 23, and 35 DAPs in PCC-1, PCC-2, PCC-7, and Mia PaCa-2, respectively, showed successive changes in their abundance corresponding to treatment time points in GemR cells, independent of the change in the control group (Table 2).

**Figure 5:**
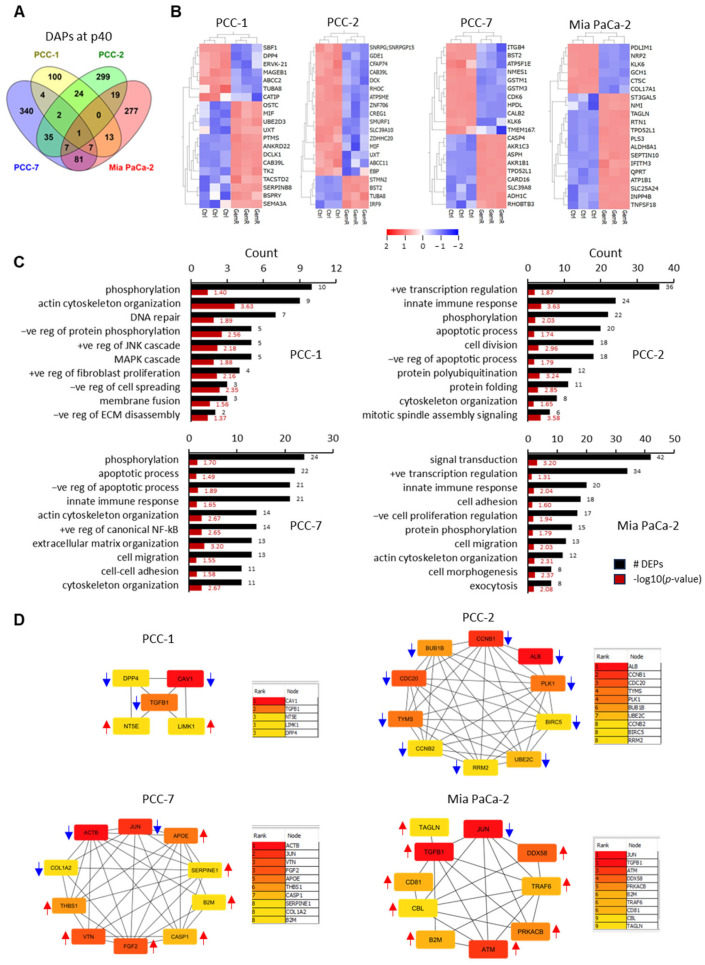
Differentially abundant proteins (DAPs) at p40. (**A**) Venn diagram showing the number of DAPs overlap between four PCC lines. (**B**) A separate heatmap of the 20 most altered DAPs in individual PCC lines. (**C**) Ten most enriched biological processes (*p* < 0.05) for each PCC line revealed by gene ontology analysis. Black and red bars indicate DAP count and the significance of enrichment, respectively. *p*-values are log10 transformed. (**D**) Connectivity between the top hub proteins identified by the cytoHubba plug-in of Cytoscape, including 5 hub proteins for PCC-1 and 10 each for the 3 other PCCs. The degree increases from yellow to light red to darker red. Red and blue arrows next to each DAP indicate their significantly higher or lower abundance in GemR versus paired control cells at p40. GemR, gemcitabine-treated cells; PCC, pancreatic cancer cell.

Third, DAPs at the last time point, p40, were investigated (shown in Figure 3A). It corresponds to 151 DAPs in PCC-1 (104↑, 47↓), 387 in PCC-2 (100↑, 287↓), 477 in PCC-7 (262↑, 215↓), and 405 in Mia PaCa-2 (251↑, 154↓). Of these, 16 DAPs were common to any 3 PCC lines. CDK6, KLK6, and PTMS were common to three primary PCCs; ATP1A3, CALB2, HDDC2, NT5E, SLC2A14, TPD52L1, and ZDHHC21 were common to PCC-1, PCC-7, and Mia PaCa-2; and HKDC1, IGF2, MALT1, MLPH, PSMB9, and SLC7A11 were common to PCC-2, PCC-7, and Mia PaCa-2 (Figure 5A, Supplementary Materials File S2). The abundance of the majority of the 20 most altered DAPs at p40 in each PCC line was higher for PCC-1 and Mia PaCa-2 and lower for PCC-2 and PCC-7 in GemR versus controls (Figure 5B). Moreover, DAPs at p40 in each PCC line were searched for the abundance of proteins known to be associated with GEM resistance mechanisms, including hENT1 (SLC29A1), DCK, CDA, DCTD, NT5E (CD73), RRM1, and RRM2. While none of these were identified as DAPs at p40 in PCC-7, a significantly higher abundance of RRM1 in PCC-1 and Mia PaCa-2, and a lower abundance of DCK and RRM2 in PCC-2, were observed. However, a clear pattern of successive change in the protein abundance along the treatment timeline was observed only for RRM1 in Mia PaCa-2 (Table 2).

### 3.6. Enrichment Analysis

DAPs at p40 (*p* < 0.05, FC > 1.2) for the four individual PCC lines were explored using gene ontology (GO) analysis for significantly enriched biological processes and KEGG pathways relevant to cancer. The ten most enriched biological processes for each PCC line included phosphorylation, transcription regulation, apoptosis, cytoskeleton organization, innate immune response, cell adhesion, and cell migration (Figure 5C). Among the enriched KEGG pathway terms, metabolic pathways were seen in all four PCC lines, followed by nucleotide metabolism, including metabolism of purines or pyrimidines, and cellular senescence in three PCC lines except PCC-1 (Table 3). Furthermore, enrichment of pathway terms, including proteoglycans in cancer, lysosomes, and C-type lectin receptor signaling were seen for both PCC-7 and Mia PaCa-2 (Table 3). Next, enrichment analysis was also performed for DAPs with significantly altered overall proteome profiles when comparing GemR versus the control group (presented in Figure 2B). The ten most enriched biological processes for each PCC line are provided in Figure S4. Among KEGG pathway terms, PCC-2 showed enrichment of pathways of neurodegeneration and motor proteins, while PCC-1 showed enrichment of pathway term cytoskeleton in muscle cells. The latter was also found enriched in both PCC-7 and Mia PaCa-2, together with several other pathways, mainly metabolism related (Table S2).

### 3.7. Protein–Protein Interaction (PPI) Networks and Identification of Hub Proteins

**Figure 6:**
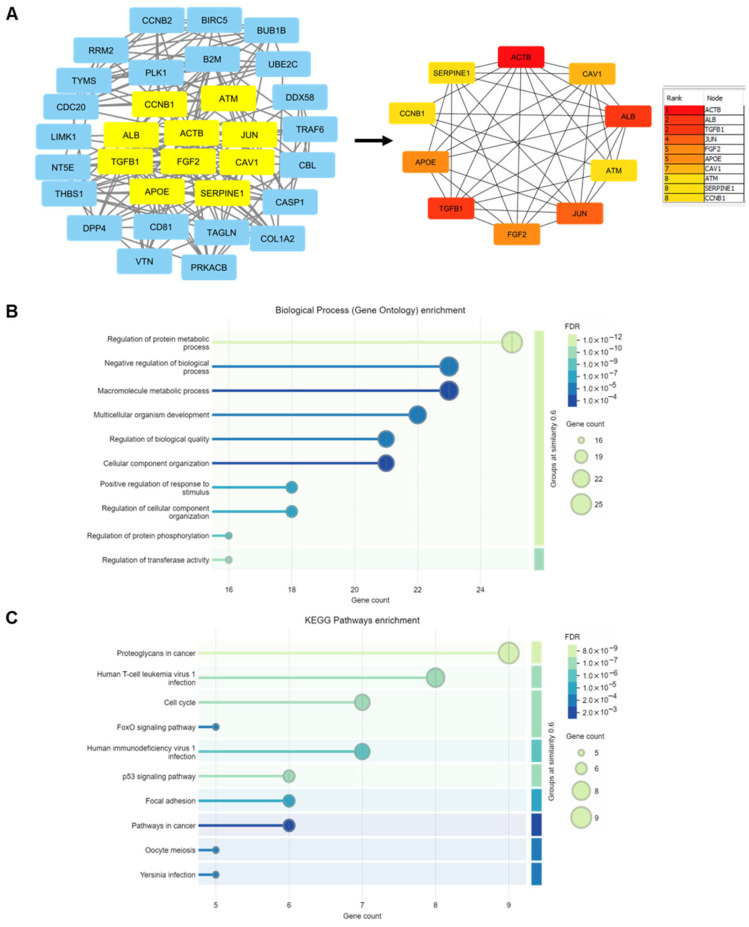
Analysis of hub proteins combined for all PCC lines. (**A**) Core protein–protein interaction (PPI) network visualized using Cytoscape. The ten proteins with higher degrees of centrality (number of links to a given node/protein) are highlighted in yellow and presented as a separate network. (**B**,**C**) Gene ontology analysis revealed the ten most enriched (*p* < 0.05) (**B**) biological processes and (**C**) KEGG pathways, arranged based on the significance of enrichment. FDR, false discovery rate; KEGG, Kyoto Encyclopedia of Genes and Genomes; PCC, pancreatic cancer cell.

DAPs at p40 (*p* < 0.05, FC > 1.2) for each PCC line were submitted to the STRING database to obtain PPI networks, which were then visualized by using Cytoscape. Core PPI networks, together with the number of nodes and edges (interactions between proteins) for each PCC line, are provided in Figure S5. Top hub proteins were selected by analyzing the topological properties and calculating the degree and betweenness centrality. Degree centrality is the number of links to a given node, while betweenness centrality measures the number of shortest paths passing through a node within a network. DAPs in the ≥95th percentile of the degree distribution of relevant networks were identified. This analysis revealed 5 proteins for PCC-1 and 10 for each of the other three PCC lines (Figure 5D). Top-ranked hub genes with the highest interaction levels included caveolin 1 (CAV1) and transforming growth factor beta-1 proprotein (TGF-β1) for PCC-1, albumin (ALB) and cyclin-B1 (CCNB1) for PCC-2, beta-actin (ACTB) and Jun proto-oncogene (JUN) for PCC-7, and JUN and TGF-β1 for Mia PaCa-2. Interestingly, all hub proteins for PCC-2 were less abundant, while most hub proteins in the other PCC lines were more abundant in GemR versus paired controls (Figure 5D). Furthermore, 35 hub proteins combined were analyzed for PPI network and GO enrichment. The core PPI network of all these hub proteins is shown in Figure 6A. Of these, the top ten proteins with the highest degree of interactions were ACTB, ALB, TGFB1, JUN, FGF2, APOE, CAV1, ATM, SERPINE1, and CCNB1 (Figure 6A). The most enriched biological processes included metabolic processes, cellular processes, component organization, and protein phosphorylation (Figure 6B). Among enriched KEGG pathways, those with known relevance to cancer were proteoglycans in cancer, cell cycle, FoxO signaling, p53 signaling, focal adhesion, and pathways in cancer (Figure 6C).

## 4. Discussion

Gemcitabine (GEM) has been pivotal to PDAC treatment strategies for nearly three decades as a monotherapy or combination regimen, both for metastatic disease and as an adjunct to surgery in operable disease [7,8,36]. However, cancer cell resistance to chemotherapy often limits treatment efficacy, leading to failure in achieving the desired outcomes [11]. The current knowledge about the mechanisms underlying the initiation and development of chemoresistance is insufficient to develop strategies to avoid and overcome this challenge. In particular, the GEM-induced molecular changes that contribute to cellular adaptation causing reduced treatment efficacy are poorly studied. To this end, the present study demonstrates comparative proteomic profiling between PCCs exposed to GEM for 40 passages (GemR group) and paired untreated controls for four PCC lines, including three primary (PCC-1, PCC-2, and PCC-7) and the established Mia PaCa-2.

The four PCC lines showed heterogenous responses to long-term exposure to GEM. Morphology, which differed between the PCCs at basal conditions, was mostly unaffected by GEM exposure. Growth was reduced in GemR versus paired controls for all PCCs at p10 and for PCC-1 and Mia PaCa-2 at p40. Consistent with previous reports, we observed reduced GEM sensitivity following long-term exposure to GEM in GemR versus paired controls for all PCCs at p40 [37,38]. Notably, all primary PCCs at p40 showed lower GEM sensitivity compared with Mia PaCa-2, in the order of PCC-2 > PCC-7 > PCC-1. Cells at p10 displayed a somewhat diverse response, with GemR versus controls showing a relatively higher sensitivity for PCC-1 and slightly less sensitivity for both PCC-7 and Mia PaCa-2. This diversity was reflected in the proteome data where the number of DAPs was markedly higher at p10 compared with p40 for PCC-1 only. Moreover, we and others have previously suggested an enhanced glycolytic phenotype in GEM-resistant PDAC cells [14,39]. Assessing extracellular lactate content as an indirect measure of glycolysis revealed no clear, consistent pattern between GemR versus controls, except for PCC-7 and Mia PaCa-2 at p10, where GemR cells appear relatively more glycolytic than paired controls.

In this study, whole-cell proteome profiles were obtained using DIA-based MS analysis. This technique has recently emerged as one of the most powerful tools for proteomic profiling, as it can map a large proteome coverage in a small sample quantity with quantitative consistency and analytic accuracy [26,27]. Using DIA-MS, Zhou et al. recently mapped an average of 7505 proteins for each of 16 major human cancer types [40], and Kong et al. mapped an average of 6668 proteins for each of 7 different human PDAC cell lines [23]. Similarly, our analysis identified an average of 7142 proteins for the four PCC lines investigated. The PCA plots revealed a notable diversity in distribution among the PCC lines in terms of overall abundance and DAPs between GemR and control groups at the individual time points (passages). Notably, the DAP count at a given time point and its pattern differed both within and among the four PCC lines. The cellular proteome profile alterations in response to GEM exposure differed between the PCCs at multiple levels. As such, a likely adaptation in response to GEM was reached by p20 in PCC-1 and by p30 in PCC-7, while both PCC-2 and Mia PaCa-2 continued to show increased differential profiles between GemR and controls up to the last time point studied.

Several recent studies have attempted to identify markers of GEM resistance in PDAC by investigating PCC lines with different GEM sensitivities [23,41] or by comparative analysis between GEM-resistant and -sensitive cells [17,25,38,42]. In the present study, proteome profiles between both groups were compared in three different ways. First, the overall proteome profile was compared between both GemR and control groups, i.e., the average of all samples in each group, independent of passage number. It identified 216, 63, 581, and 165 DAPs in PCC-1, PCC-2, PCC-7, and Mia PaCa-2, respectively, while none overlapped between the four PCC lines. Of these, only ASB3, RPL22L1, and UFM1, which have been reported for their role in the progression of various cancers, were common across the three primary PCC lines; however, their contribution to chemoresistance remains unknown. It has been reported that ASB3 and UFM1 are involved in regulation of ubiquitination, a key post-translational modification [43,44], while RPL22L1 regulates ribosome assembly and protein synthesis [45]. Second, the top 100 DAPs in each PCC line identified through multi-group comparison were examined for passage-associated proteome alterations, as these could possibly be impacted by longer passaging [46,47]. The abundance of several DAPs was unchanged in the control group throughout 40 passages, and most of these were upregulated passage-dependently in the GemR group.

Lastly, we examined DAPs at p40, the longest exposure time point, which made up 2.1, 5.4, 6.7, and 5.7% of the average number of proteins identified in PCC-1, PCC-2, PCC-7, and Mia PaCa-2, respectively. The p40 DAPs belong to diverse biological processes, including phosphorylation, transcription regulation, apoptosis, cytoskeleton organization, innate immune response, cell adhesion, and cell migration. Moreover, enrichment of KEGG pathway term metabolic pathways was seen in all four PCC lines, followed by metabolism of nucleotides, cellular senescence, proteoglycans in cancer, lysosomes, and C-type lectin receptor signaling, which was present in at least two of the PCC lines studied. Recent reports from our group [14,32] and others [17,48,49,50] have revealed an association between drug resistance and cancer metabolism in PDAC. The PPI network analysis of DAPs at p40 identified several hub proteins, including top-ranked CAV1, TGF-β1, ALB, CCNB1, ACTB, and JUN. It has been reported that GEM increases the expression of ALB transporter protein CAV1, which is associated with PDAC progression and drug resistance phenotype [51,52]. TGFβ, which has both protumor and antitumor effects, depending on the pancreatic TME, is known to be a GEM resistance-associated gene [53,54,55]. As an important cell cycle regulating protein, CCNB1 has also been reported to have an oncogenic role in several human tumors [56], which upon silencing, promotes senescence and inhibits proliferation [57]. Similarly, the proto-oncogene Jun has been shown to be a driver of cancer cell metabolic reprogramming [58] and to be associated with GEM resistance in PDAC [59].

The proteomic approach is increasingly used to identify potential makers of GEM resistance in PDAC [17,23,25,42]. However, none of these studies investigated the proteome changes associated with the duration of GEM exposure. To the best of our knowledge, this is the first study to investigate and report the proteomic profile of three primary human PCCs as well as the well-established cell line Mia PaCa-2 at six subsequent time points during their exposure to GEM for 40 passages, equivalent to a total treatment duration of 7 to 8 months. In addition to heterogeneity in phenotypic characteristics, overall proteome profiles as well as the pattern of change in cellular proteomes in response to GEM were found to be markedly different when comparing the four PCC lines. Most GEM-induced changes in proteome profiles were seen in the first half of the treatment timeline for PCC-1 and PCC-7, whereas they were in the latter half for PCC-2 and Mia PaCa-2. The abundance of several proteins at p40 differed between GemR and paired controls, which could be considered potential markers of GEM response. However, all these p40 DAPs were cell-line-specific, with none being common to all four PCCs, and their expression patterns were affected by the duration of treatment. Most p40 DAPs were related to biological processes of cell growth and metabolic pathways, including the metabolism of nucleotides. Augmented nucleotide metabolism is a critical metabolic dependency of cancer cells that has been implicated in chemotherapy resistance. A molecular competition between endogenous nucleotides and nucleoside analog chemotherapy drugs has been suggested to affect cytotoxicity [60]. Likewise, as the GEM metabolite in its tri-phosphate form (dFdCTP), which exerts toxicity, competes with endogenous deoxycytidylate (dCTP), the proportion of GEM-induced toxicity is related to the ratio of dFdCTP to endogenous dNTPs in target cells. Taken together, these findings reveal that the GEM-induced changes in the cellular proteome are cell-line-specific and suggest that the presence of profound heterogeneity among PCCs may impede straightforward identification of potential markers of GEM resistance in PDAC.

This study demonstrates the strengths of proteomic analysis in discovering potential treatment-induced changes in PDAC. The results of this study provide a foundation for further research to validate and expand upon these findings, using larger sample sizes of primary cells, tumor-derived organoids, or serum samples. Moreover, neoadjuvant chemotherapy has been increasingly used in recent years in the treatment of borderline and locally advanced PDACs; however, the exploitation of its full benefits is limited due to a lack of specific treatment response markers. To this end, the proteomic approach used in this study could be beneficial. Analysis of tumor samples (fine-needle biopsy) and matched serum samples collected on several occasions during the treatment timeline for individual PDAC patients undergoing neoadjuvant treatment appears to be an ideal approach. As such, assessing the individual patient’s response to chemotherapy could help in identifying individuals who respond to a given treatment. In addition, this could contribute to revealing treatment-induced molecular changes in the tumor and at the systemic level to identify potential markers of treatment response. Lastly, such analysis may be a step toward personalized medicine.

While this study successfully identified several DAPs using DIA-based proteomics, we acknowledge that protein abundance alone does not fully reflect protein activity and that the identification of proteoforms is important to better understand the complexity of biological systems, disease mechanisms, and therapeutic vulnerabilities [61]. Proteoforms are the structurally and functionally distinct protein species arising from alternative splicing, post-translational modifications, and genetic variation. Identification of proteoforms requires modification of MS workflows and/or integration with other techniques like genomics analysis [62,63], which is beyond the scope of this study.

This study has certain limitations. First, the primary PCCs used in this study may not be as stable as Mia PaCa-2, as long-term culturing may alter their growth pathways differentially. Second, complete loss of GEM sensitivity may not have been achieved in all PCC lines at p40. Lastly, proteomics data would require further experimental validation, including functional characterization, to identify potential markers of GEM resistance in PDAC.

## 5. Conclusions

The cellular adaptations in response to long-term GEM exposure were markedly different among the four PCC lines. The heterogeneity was visible at multiple levels, including phenotypic characteristics as well as overall and passage-associated differences in proteome profiles. Differentially abundant proteins between GEM-treated and control cells were cell-line-specific, and most of these were related to metabolic pathways, including the metabolism of nucleotides. The results suggest that identification of unique markers of GEM resistance in PDAC appears to be complicated by the significant heterogeneity among PCCs and by the effects of treatment duration, dosage, and passaging on protein abundance.

## Figures and Tables

**Table 1:** DAPs between control and GemR cells common to different PCC lines.

Gene (Protein ID)	Description	Molecular Function	Biological Process
DAPs common to all four PCC lines			
KLK6 (Q92876)	Kallikrein-6	Hydrolase, protease	CNS development, collagen catabolism, differentiation, invasion
DAPs common to three primary PCC lines			
AFP (P02771)	Alpha-fetoprotein	Metal-ion binding	Ovulation, progesterone metabolic process
ALG5 (Q9Y673)	Dolichyl-phosphate beta-glucosyltransferase	Glycosyl-transferase	Glycosylation
ASB3 (Q9Y575)	Ankyrin repeat and SOCS box protein 3	Protein modification	Protein ubiquitination
C1QTNF3 (Q9BXJ4)	Complement C1q tumor necrosis factor-related protein 3	Identical protein binding	Cellular triglyceride homeostasis, NF-kB signaling, cytokine production, gluconeogenesis
MT-ATP6 (P00846)	ATP synthase subunit a	Transmembrane transporter	ATP synthesis, ion transport
NDUFA4 (O00483)	Cytochrome c oxidase subunit NDUFA4	NADH activity	Electron transport, respiration
NUFIP1 (Q9UHK0)	Nuclear fragile X mental retardation-interacting protein 1	RNA-binding	Transcription regulation, RNA processing
PRR14L (Q5THK1)	Protein PRR14L	Unknown	Cell division, skeletal myogenesis, tumorigenesis
TMA7 (Q9Y2S6)	Translation machinery-associated protein 7	Cytoplasmic translation	Tumor progression, proliferation
TNIK (Q9UKE5)	TRAF2 and NCK-interacting protein kinase	Kinase activity, transferase	Neurogenesis, Wnt signaling pathway
UFM1 (P61960)	Ubiquitin-fold modifier 1	Ubiquitylation	Apoptosis, ufmylation, Ubl conjugation pathway
UXT (Q9UBK9)	Protein UXT	Chaperone	Apoptosis, transcription regulation, chromatin/microtubule binding
DAPs common to PCC-2, PCC-7, and Mia PaCa-2			
CCDC71L (Q8N9Z2)	Coiled-coil domain-containing protein 71L	Lipid metabolic process	Fat cell differentiation
IGFBP6 (P24592)	Insulin-like growth factor-binding protein 6	Growth factor binding	Cell growth, migration, signal transduction, MAPK cascade
INPP4B (O15327)	Inositol polyphosphate 4-phosphatase type II	Hydrolase	Lipid metabolism
MALT1 (Q9UDY8)	Mucosa-associated lymphoid tissue lymphoma translocation protein 1	Hydrolase, protease	Inflammatory response, immunity, Ubl conjugation pathway
PSMB9 (P28065)	Proteasome subunit beta type 9	Hydrolase, protease	Host-virus interaction, immunity, endopeptidase activity
TGM2 (P21980)	Protein-glutamine gamma-glutamyltransferase 2	Acyltransferase, hydrolase	Apoptotic process, inflammation, cell adhesion

Differentially abundant proteins (DAPs) between control and GemR cells that were common to different pancreatic cancer cell PCC lines—PCC-1, PCC-2, PCC-7, and Mia PaCa-2—were searched. The information about their reported molecular function and involvement in biological processes was obtained from the public databases UniProt and GeneCards.

**Table 2:** DAPs with altered levels during subsequent GEM exposure.

Cell Line	Change	List of Proteins
Altered expression in GemR versus ‘unchanged control’		
PCC-1	↑	ACACA, ALDH1A3, CAPN1, CKAP4, CLIP1, DPYSL2, DUSP4, DYNC1H1, ENO2, GDA, GSN, IQGAP1, KRT19, LAMB3, MACF1, PGD, PKM, PPP1R12A, SERPINB5, VCAN, VPS13C
	↓	ELAC2, FABP5, FLNC, HECTD1, MANF, MPI, PFKP, PTK2, RRBP1, UBE2S
PCC-2	↑	BST2, DECR1, MBOAT7, PPL, RUFY1, SAMD9, TKFC, TM7SF2, TRIM21
	↓	DNMT1, EZR, HMMR, MALT1, MCAM, NSUN2, PBK, SDHA, SNRPG, TACC3, TMA7, TTK, UFM1, UXT
PCC-7	↑	AKR1B1, AKR1C1, ALDH1A3, GBP2
	↓	ATAD3B, CKAP4, DENND5B, EPS8, FAM114A1, FDXR, GOLGA3, GUSB, HPDL, NT5E, ORC6, P3H3, RRP1B, SEPTIN10, SLC4A2, SVIL, UACA
Mia PaCa-2	↑	ARHGEF12, ATM, CARS1, COL3A1, CTNND1, DCPS, PTPRF, RDX, RRM1, TNKS1BP1, UTRN
	↓	DOCK11, FDXR, IDH1
Passage-associated successive change in expression in GemR group		
PCC-1	↑	ALDH16A1, ARFIP2, CD109, CKAP4, MID1, MPI, MTUS1, PGD, PPP1R12A, SQOR
	↓	PDP1, PFKP
PCC-2	↑	MACROD1, MAOB, MBOAT7, PPL, SAMD9, SQSTM1, TM7SF2, TUBA8
	↓	ANLN, ASB3, AURKA, CCT3, CCT5, CCT7, CFAP74, CLPTM1L, DNMT1, EZR, FAM92A, GOLPH3, HMMR, ITGA6, KIF2C, MALT1, MTRR, NSUN2, NUP155, PBL, PLK1, RBM23, SNRPG, TACC3, TARS1, TMA7, TTK, UFM1, UXT, ZNF622
PCC-7	↑	AK3, ASPH, FBXO2, FMNL2, HKDC1, NAMPT, NBAS, NT5E, PDP1, PSMB8, SEC62, SLC22A18, SYTL2, TPD52L1
	↓	ACOT7, ANXA6, ATAD3B, DPYD, FAM114A1, FDXR, FKBP5, HPDL, PDCD4
Mia PaCa-2	↑	AKR1B1, CARS1, DCPS, EPHA4, FHL1, MALT1, RNH1, RRM1, SEPTIN10, SWAP70, TALDO1, TRIM21
	↓	ANXA2, ATP13A3, DOCK11, DPYD, IDH1, ITPR3, KRT80, MYOF, NAMPT, NQO2, PDLIM1, PLEC, PODXL, PYGB, RAP2B, SERPINB6, SLC25A13, SNCG, STAT3, TP63, TRIP12, UAP1, YWHAQ

Differentially abundant proteins (DAPs) in GemR versus controls showing a successive passage-dependent change in abundance in GemR cells. Arrows indicate the direction of change in expression from p10 toward p40.

**Table 3:** Enriched KEGG pathways for DAPs at p40.

Cell Line	Pathway ID	Description	Count	%	*p*-Value	Adj. *p*-Value
PCC-1	hsa01100	Metabolic pathways	25	16.6	<0.01	0.03
	hsa00240	Pyrimidine metabolism	6	4.0	<0.01	0.02
	hsa01232	Nucleotide metabolism	6	4.0	<0.01	0.03
	hsa01240	Biosynthesis of cofactors	5	3.3	<0.05	1.00
PCC-2	hsa01100	Metabolic pathways	42	10.9	<0.05	1.00
	hsa04110	Cell cycle	15	3.9	<0.01	0.00
	hsa04120	Ubiquitin mediated proteolysis	11	2.8	<0.01	0.07
	hsa04218	Cellular senescence	9	2.3	<0.01	0.60
	hsa01232	Nucleotide metabolism	8	2.1	<0.01	0.14
	hsa00240	Pyrimidine metabolism	6	1.6	<0.01	0.32
	hsa03420	Nucleotide excision repair	5	1.3	<0.04	1.00
PCC-7	hsa01100	Metabolic pathways	61	12.8	<0.01	0.06
	hsa05200	Pathways in cancer	22	4.6	<0.05	0.47
	hsa05205	Proteoglycans in cancer	17	3.6	<0.01	0.01
	hsa04151	PI3K-Akt signaling pathway	17	3.6	<0.05	0.37
	hsa04510	Focal adhesion	15	3.1	<0.01	0.06
	hsa04512	ECM-receptor interaction	10	2.1	<0.01	0.05
	hsa04218	Cellular senescence	10	2.1	<0.05	0.33
	hsa04142	Lysosome	9	1.9	<0.05	0.33
	hsa04625	C-type lectin receptor signaling pathway	8	1.7	<0.05	0.33
	hsa04066	HIF-1 signaling pathway	8	1.7	<0.05	0.33
Mia PaCa-2	hsa01100	Metabolic pathways	55	13.6	<0.01	0.05
	hsa04010	MAPK signaling pathway	16	4.0	<0.01	0.07
	hsa05205	Proteoglycans in cancer	15	3.7	<0.01	0.03
	hsa04022	cGMP-PKG signaling pathway	14	3.5	<0.01	0.02
	hsa04024	cAMP signaling pathway	11	2.7	<0.05	0.27
	hsa04210	Apoptosis	10	2.5	<0.01	0.07
	hsa04218	Cellular senescence	10	2.5	<0.01	0.14
	hsa04625	C-type lectin receptor signaling pathway	9	2.2	<0.01	0.06
	hsa04142	Lysosome	9	2.2	<0.01	0.14
	hsa00230	Purine metabolism	8	2.0	<0.05	0.24

Differentially abundant proteins (DAPs) between GemR versus controls at p40 (*p* < 0.05, FC > 1.2) for each PCC line were searched for the enrichment of KEGG pathways using David, the Database for Annotation, Visualization, and Integrated Discovery. The above list includes all or the 10 most enriched pathways (*p* < 0.05) for each PCC line with known relevance to cancer. PCC, pancreatic cancer cell.

## Data Availability

The datasets generated and/or analyzed during the study are available in the ProteomeXchange Consortium via Proteomics Identification Database (PRIDE) repository with the dataset identifier PXD063320.

## Supplementary Materials

The following supporting information can be downloaded at: https://www.mdpi.com/article/10.3390/proteomes13040048/s1, File S1: Complete proteomics data. File S2: List of DAPs at individual time points in each PCC line. Figure S1: Heatmaps of DAPs common to all six time points. Figure S2: DAPs overlapping between PCC lines. Figure S3: Expression profiles detected by proteomics and full blot images for immunoblotting. Figure S4: Enriched biological processes for DAPs with overall change between both groups. Figure S5: Protein–protein interaction networks for p40 DAPs. Table S1: DAPs with unchanged levels in the control group throughout 40 passages. Table S2: Enriched KEGG pathways for DAPs with significantly altered overall proteome profiles.

## Abbreviations

The following abbreviations are used in this manuscript:

DAPs	Differentially abundant proteins
FC	Fold change
GemR	Gemcitabine-treated cells
GO	Gene ontology
KLK6	Kallikrein-6
MALT1	Mucosa-associated lymphoid tissue lymphoma translocation protein 1
MS	Mass spectrometry
NGM	Normal growth medium
PCA	Principal component analysis
PCC	Pancreatic cancer cell
PDAC	Pancreatic ductal adenocarcinoma
PPI	Protein–protein interaction
