# Supplementary material for: Proteomic Characterization of Primary Human Pancreatic Cancer Cell Lines Following Long-Term Exposure to Gemcitabine
Source: Proteomes. 2025 Oct 1;13(4):48. doi: 10.3390/proteomes13040048 (PMC12551111; doi:10.3390/proteomes13040048)
Supplement: Supplementary file 1 [file proteomes-13-00048-s001.zip › Supplementary Files/Figures S1-S5.pdf]

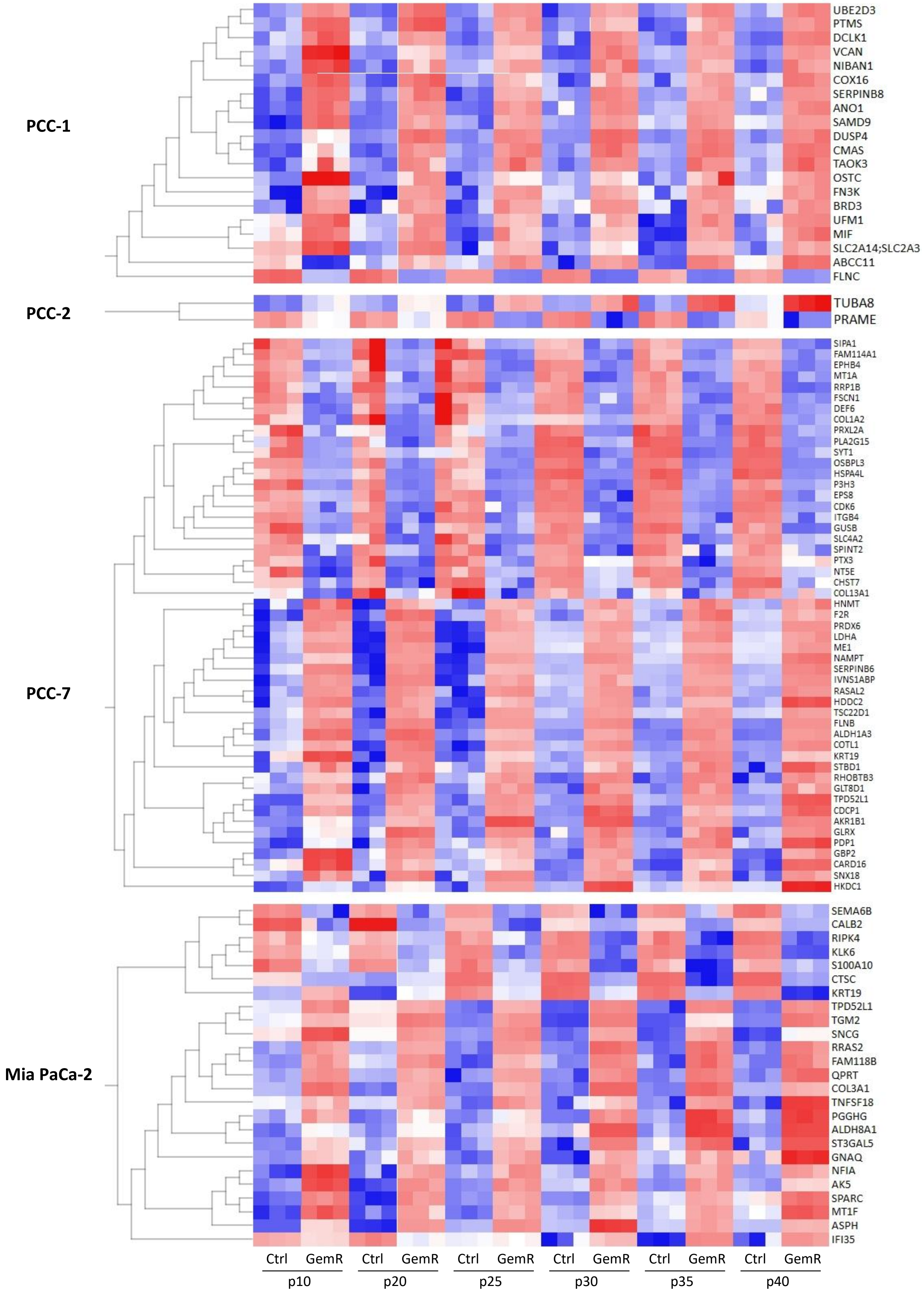

Figure S1. Heatmaps of DEPs common to all six time points in the individual pancreatic cancer cell (PCC) line. Ctrl, control cells; GemR, gemcitabine resistant cells; p, passage.

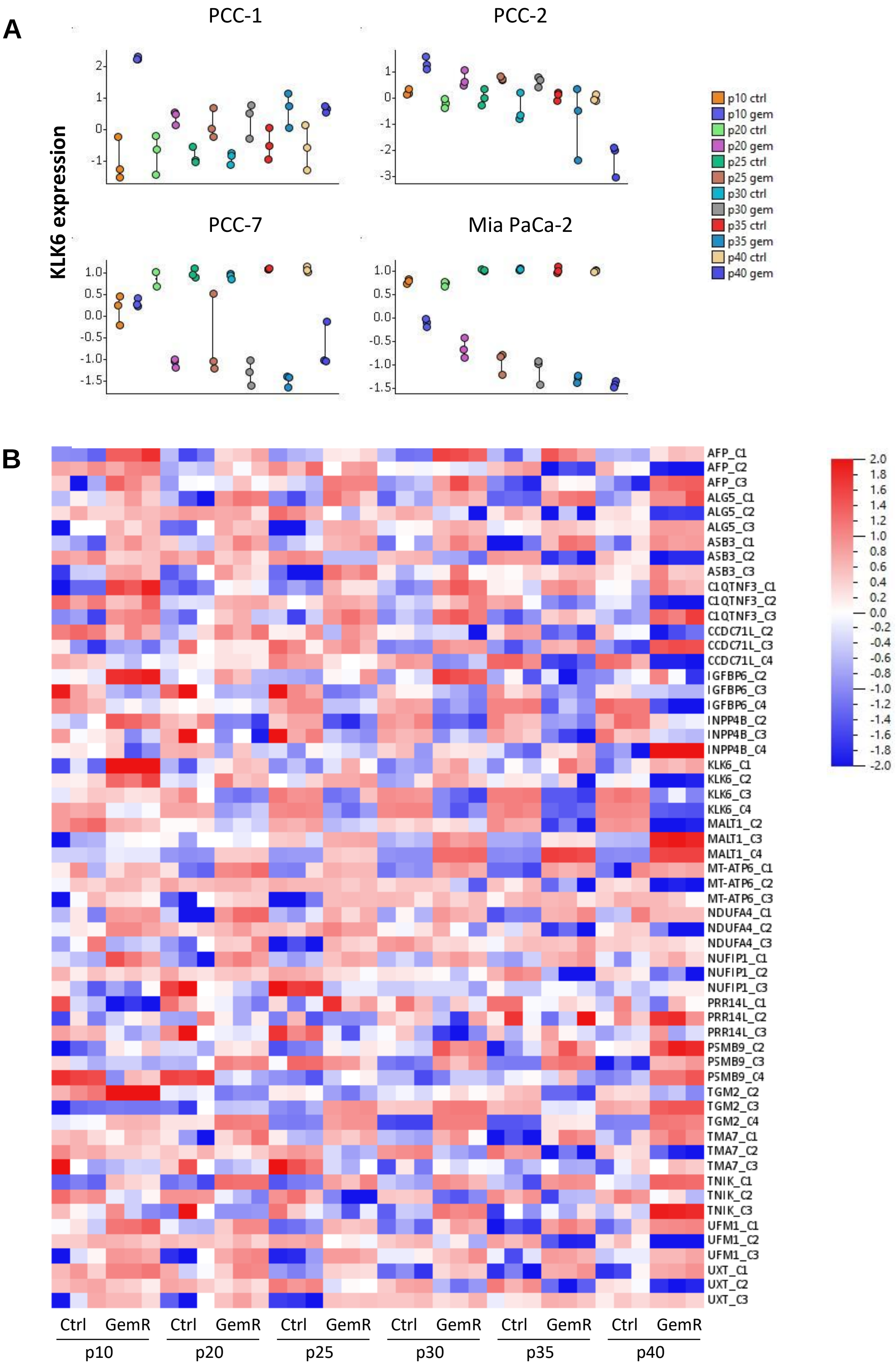

Figure S2. DEPs overlapping between four pancreatic cancer cell (PCC) lines. **A**. Expression patten of KLK6 (Kallikrein-6) across four PCC lines at all time points. **B**. Heatmap showing expression profiles of DEPs common to different PCCs. In **B**, C1, C2, C3, and C4 corresponds to PCC-1, PCC-2, PCC-7 and Mia PaCa-2, respectively. Ctrl, control cells; gem, GemR, gemcitabine resistant cells; p, passage.

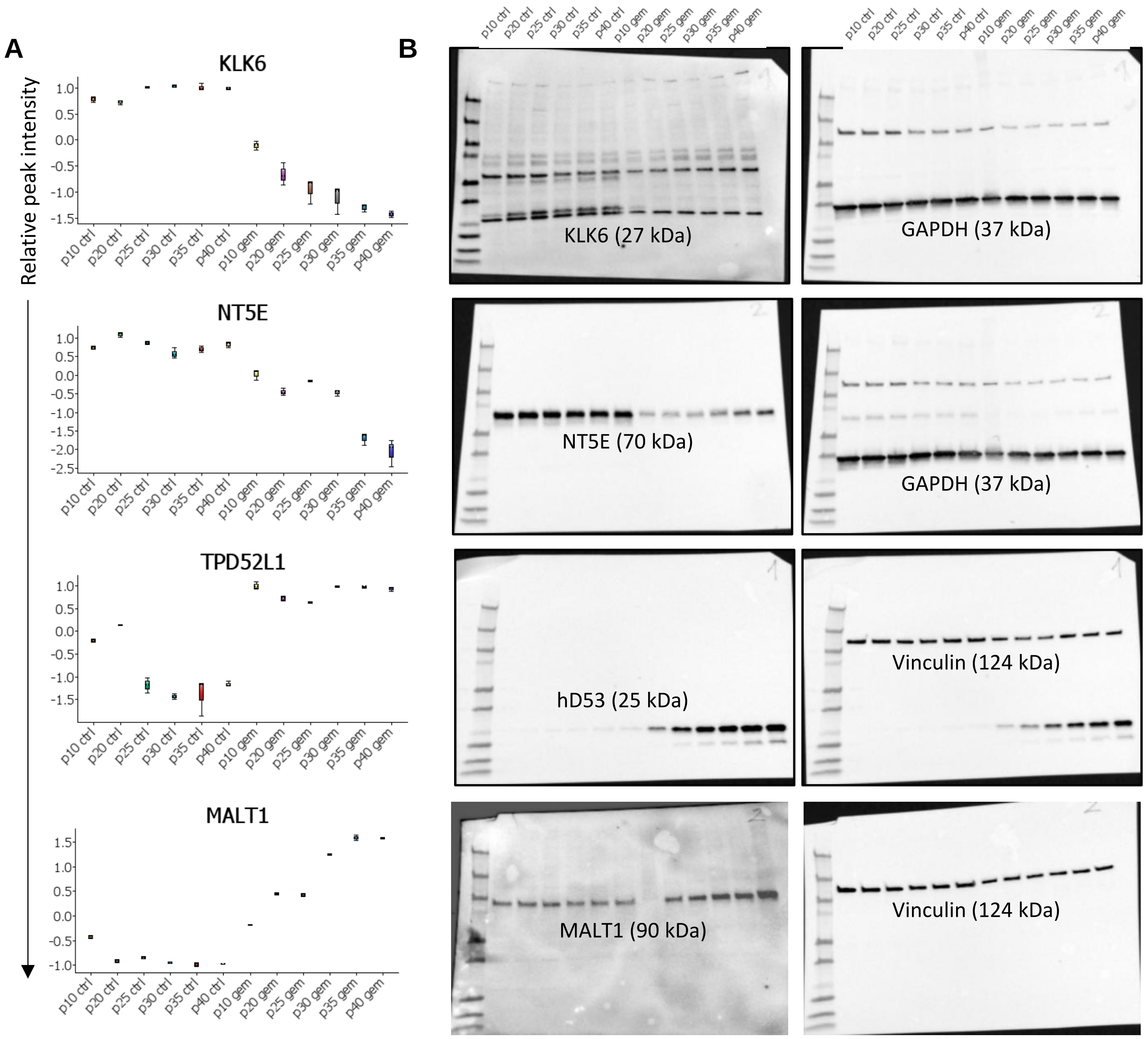

Figure S3. Validation of proteomic data. **A.** Expression profiles by proteomics analysis. **B.** Full blot image of the representative blots presented in Fig. 3C showing expression of KLK6, NT5E, hD53, and MALT1. GAPDH and Vinculin were used as loading controls. GAPDH, glyceraldehyde-3-phosphate dehydrogenase; KLK6, kallikrein-6; MALT1, mucosa-associated lymphoid tissue lymphoma translocation protein 1; NT5E, 5'-nucleotidase; TPD52L1 (hD53), tumor protein D53.

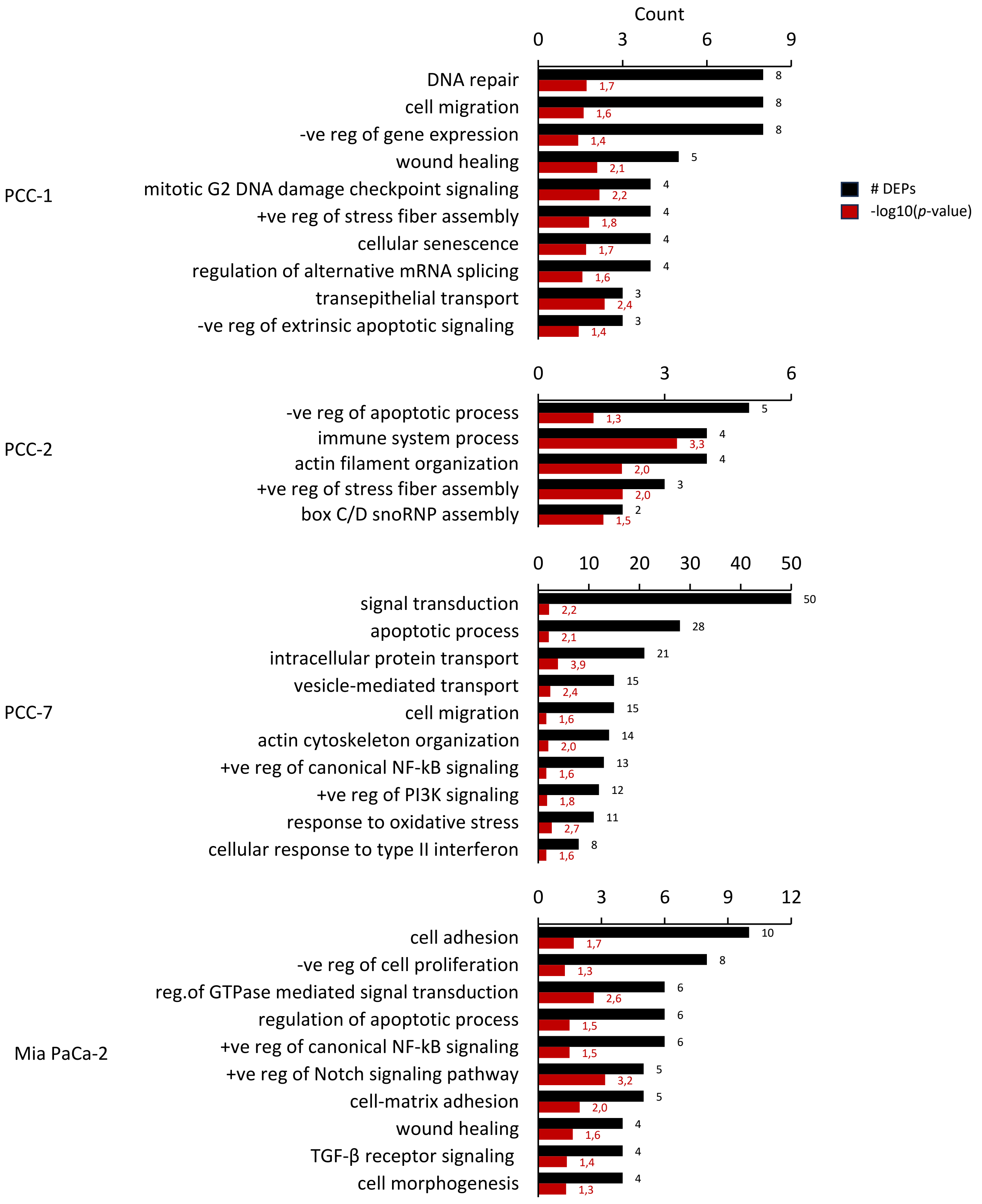

Figure S4. Up to 10 most enriched biological processes ($p<0.05$) for DEPs with significantly altered overall proteome profile between GemR versus control cells for each PCC line by gene ontology analysis. Black and red bars indicate DEP count and the significance of enrichment, respectively. p-values are log10 transformed.

PCC-1

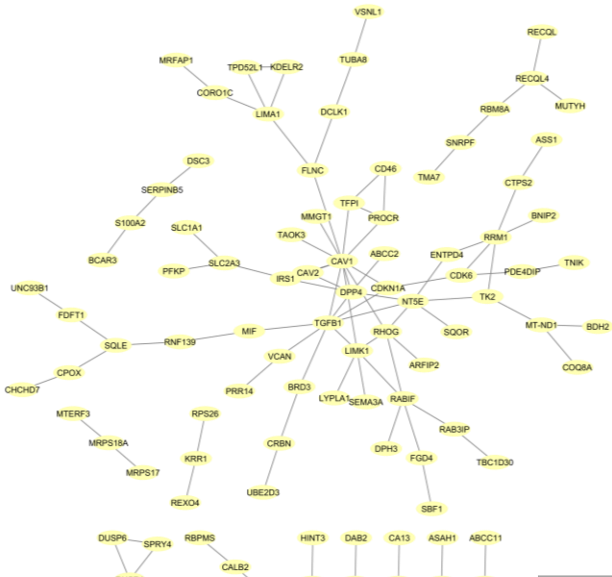

PCC-2

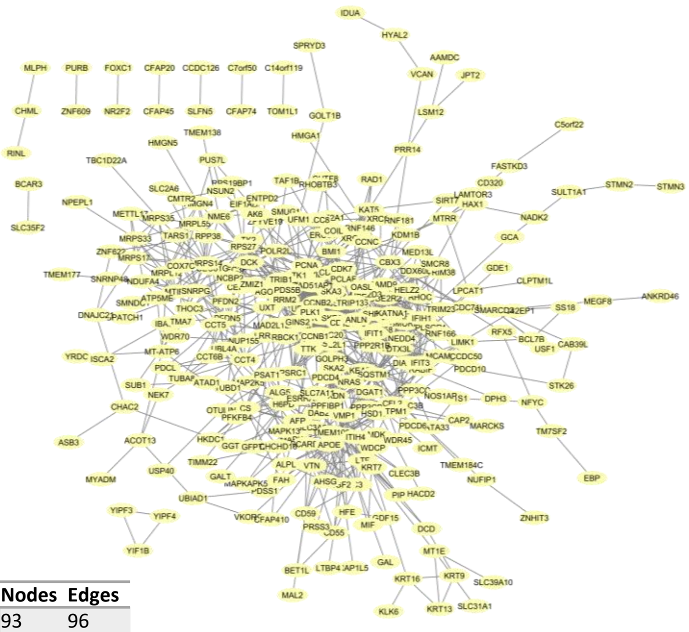

Cell line	Nodes	Edges
PCC-1	93	96
PCC-2	312	1116
PCC-7	391	1264
Mia PaCa-2	301	784

PCC-7

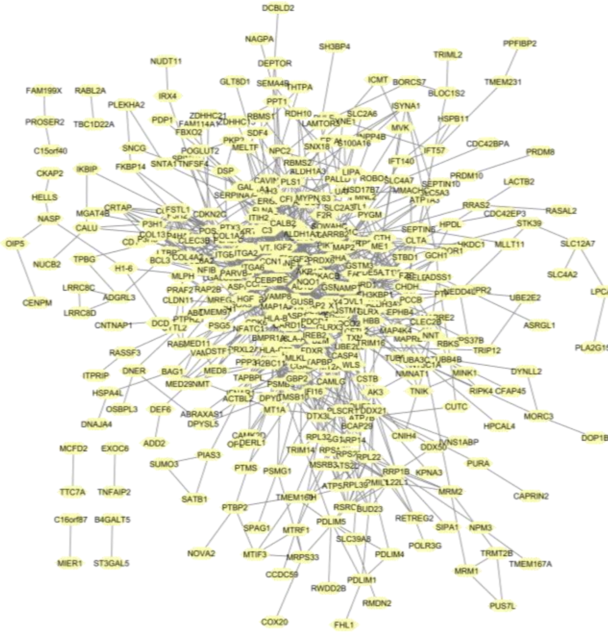

Mia PaCa-2

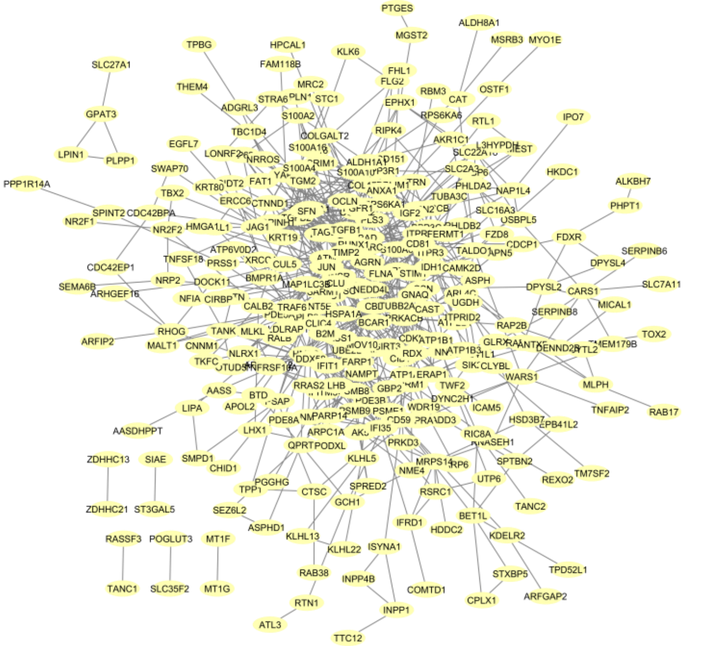

Figure S5. Core protein-protein interactions (PPI) networks for DEPs at p40 for each individual PCC line. The number of nodes and edges represents proteins and the interactions between proteins, respectively. PCC, pancreatic cancer cell.
