# Supplementary material for: Proteomic Characterization of Primary Human Pancreatic Cancer Cell Lines Following Long-Term Exposure to Gemcitabine
Source: Proteomes. 2025 Oct 1;13(4):48. doi: 10.3390/proteomes13040048 (PMC12551111; doi:10.3390/proteomes13040048)
Supplement: Supplementary file 1 [file proteomes-13-00048-s001.zip › Supplementary Files/Tables_ S1-S2.pdf]

Table S1. DEPs with expression unchanged in the control group throughout 40 passages.

PCC-1			PCC-2			PCC-7			Mia PaCa-2		
No change	↑	↓	No change	↑	↓	No change	↑	↓	No change	↑	↓
ACACA	ACO1	ALDH2	AFP	ABCF2	ASB3	AKR1B1	CDCP1	ASPH	ARHGEF12	AKR1B1	ACADM
ACSL5	ALDH16A1	CDK6	ALDH1A3	AKAP12	CD55	AKR1C1	CDK6	CARD16	ATM	AKR1C1	AHNAK2
AHNAK	ARFIP2	DOCK5	ANLN	CCT3	CFAP74	ALDH1A3	DDX21	CAVIN2	CARS1	ARHGAP18	AIP
ALDH1A3	CD109	FLNA	AURKA	CCT5	IGF2	ATAD3B	DPYSL2	DPYD	COL3A1	ARHGAP5	ALDH1A1
ANKRD22	CPNE3	GFPT1	BST2	CCT7	ITGA6	CKAP4	F2R	FKBP10	CTNND1	ASPH	ALDH7A1
CAPN1	CPOX	HK2	CBX3	CDH1	ITGB4	DENND5B	FBXO2	FMNL2	DCPS	ATP13A3	ANXA2
CKAP4	FAT1	HSP90AA1	COL12A1	CLPTM1L	MACROD1	EPS8	FKBP5	OPLAH	DOCK11	CTSC	ANXA6
CLIP1	FKBP10	HSPH1	DECR1	EPPK1	MAOB	FAM114A1	HKDC1	PCNT	FDXR	DST	ATP1B1
DPYSL2	ITGA6	NLE1	DNMT1	EPS8	MISP	FDXR	HSPA4L	PDLIM5	IDH1	ENC1	CBL
DUSP4	ITGB1	OSBPL8	EPHA2	FAM92A	PAAF1	GBP2	IQGAP2	PITRM1	PTPRF	FASN	CKAP5
DYNC1H1	ITGB4	PDLIM5	EZR	FAT1	PDLIM5	GOLGA3	LACTB2	PPP3CA	RAPH1	FERMT1	COL18A1
ELAC2	LLGL2	PDP1	HMMR	FERMT2	PLEC	GUSB	MLLT11	PSMB8	RDX	HSPA1A	CUL5
ENO2	MID1	SMC2	KPNA2	GOLPH3	RBM23	HPDL	NAMPT	PTK7	RRM1	ITPR3	DPYD
FABP5	MTUS1	SMC4	KRT7	HSPH1	SERPINB6	NT5E	PDP1	RAI14	TNKS1BP1	KRT80	DPYSL2
FDFT1	MVP	VASP	LIMA1	KIF2C		ORC6	PHGDH	SCLY	UTRN	NAMPT	ECE1
FKBP4	MYO5A		MALT1	MSH6		P3H3	PLS1	SUMF2		NNT	EPB41L2
FLNC	OAS3		MBOAT7	MTRR		RRP1B	RAB27B	TP53		NQO2	EPHA4
GDA	PROM1		MCAM	NUP155		SEPTIN10	SEC62			PDE5A	FHL1
GSN	RPS6KA3		MSH2	OAS3		SLC4A2	SH3KBP1			PDLIM1	GANAB
HECTD1	SMARCA1		NSUN2	OXCT1		SVIL	SLC22A18			PLEC	HYOU1
IQGAP1	SQOR		PBK	PLK1		UACA	SPTBN1			PODXL	ITGA3
KRT19	SRPRA		PPL	PPFIBP1			TANGO6			PYGL	MALT1
LAMB3	SYNE2		RUFY1	PPP2R1B			TARS1			SERPINB6	MVP
MACF1	TLN1		SAMD9	S100A14			TPD52L1			SLC25A13	MYOF
MANF	TOMM34		SDHA	SQSTM1						SLC4A7	NUMA1
MPI	UGP2		SNRPG	TARS1						TP63	PC
PFKP	VWA8		TACC3	TBC1D9B						VARS1	PLS3
PGD			TJP1	TRIP13							POGLUT3
PKM			TKFC	TUBA8							PYGB
PPP1R12A			TM7SF2	WDR70							RAP2B
PTK2			TMA7	ZNF622							RNH1
RRBP1			TRIM21								SCRN1
S100A4			TTK								SEPTIN10
SERPINB5			UFM1								SLK
SLC38A2			UXT								SNCG
UBE2S											STAT3
VCAN											SWAP70
VPS13C											TALDO1
AAMP											TGM2
											TRIM21
											TRIP12
											UAP1
											YWHAQ

Distribution of DEPs according to their time point associated change in expression in the control group. The direction of change in the expression corresponds to the change from p10 towards p40. DEP, differentially expressed protein; PCC, pancreatic cancer cell.

Table S2. Enriched KEGG pathways for DEPs with significantly altered overall proteome profiles.

Cell line	Pathway ID	Description	Count	%	*p*-value	adj. *p*-value
PCC-1	hsa04820	Cytoskeleton in muscle cells	8	3,7	0,02	1,00
PCC-2	hsa05022	Pathways of neurodegeneration	5	8,1	0,04	1,00
	hsa04814	Motor proteins	4	6,5	0,02	1,00
PCC-7	hsa01100	Metabolic pathways	70	12,0	0,01	0,29
	hsa04144	Endocytosis	20	3,4	0,00	0,27
	hsa05208	Chemical carcinogenesis - ROS	16	2,8	0,01	0,29
	hsa04820	Cytoskeleton in muscle cells	14	2,4	0,05	0,60
	hsa04218	Cellular senescence	13	2,2	0,01	0,29
	hsa04510	Focal adhesion	13	2,2	0,04	0,56
	hsa05205	Proteoglycans in cancer	13	2,2	0,04	0,56
	hsa04530	Tight junction	12	2,1	0,03	0,47
	hsa03013	Nucleocytoplasmic transport	11	1,9	0,00	0,29
	hsa04142	Lysosome	11	1,9	0,01	0,33
	hsa01232	Nucleotide metabolism	8	1,4	0,02	0,44
	hsa00270	Cysteine and methionine metabolism	7	1,2	0,01	0,29
	hsa01524	Platinum drug resistance	7	1,2	0,04	0,56
	hsa04216	Ferroptosis	6	1,0	0,01	0,33
	hsa00620	Pyruvate metabolism	6	1,0	0,02	0,42
	hsa00480	Glutathione metabolism	6	1,0	0,05	0,56
Mia PaCa-2	hsa04022	cGMP-PKG signaling pathway	8	4,8	0,00	0,07
	hsa05205	Proteoglycans in cancer	8	4,8	0,00	0,07
	hsa04820	Cytoskeleton in muscle cells	8	4,8	0,01	0,08
	hsa04972	Pancreatic secretion	6	3,6	0,00	0,07
	hsa04922	Glucagon signaling pathway	6	3,6	0,00	0,07
	hsa04971	Gastric acid secretion	5	3,0	0,01	0,08
	hsa04911	Insulin secretion	5	3,0	0,01	0,10
	hsa04625	C-type lectin receptor signaling pathway	5	3,0	0,02	0,18
	hsa04974	Protein digestion and absorption	5	3,0	0,02	0,18
	hsa00760	Nicotinate and nicotinamide metabolism	3	1,8	0,05	0,43

Differentially expressed proteins (DEPs) between GemR versus control cells with significantly altered overall proteome profile ($p<0.05$) for each PCC line were searched for the enrichment of KEGG pathways using the online Database for Annotation, Visualization and Integrated Discovery (DAVID). Above list includes all significantly enriched pathways ($p<0.05$) for each PCC line with relevance to cancer. PCC, pancreatic cancer cell.
